# Effects of Dexamethasone on Cognitive Functions After Coronary Artery Bypass Grafting Surgery

**DOI:** 10.3390/medicina62010011

**Published:** 2025-12-20

**Authors:** Tadas Umbrasas, Milda Švagždienė, Judita Andrejaitienė, Greta Kasputytė

**Affiliations:** 1 The Hospital of the Lithuanian University of Health Sciences Kaunas Clinics, 50161 Kaunas, Lithuania; 2 Institute of Cardiology, Medical Academy, Lithuanian University of Health Sciences, 50162 Kaunas, Lithuania; 3 Department of Anesthesiology, Medical Academy, Lithuanian University of Health Sciences, 50162 Kaunas, Lithuania

**Keywords:** dexamethasone, anesthesia, cardiac surgery, cognitive functions

## Abstract

*Background and Objectives:* Coronary artery bypass grafting (CABG) is one of the most common cardiac surgeries worldwide. However, postoperative cognitive decline (POCD) remains a significant concern, affecting a substantial proportion of patients. One of the pathogenic mechanisms underlying POCD involves inflammatory responses and oxidative stress. Dexamethasone, a corticosteroid with potent anti-inflammatory properties, has been proposed as a potential neuroprotective agent. This study aimed to assess the effect of a single perioperative dose of dexamethasone on postoperative cognitive function in patients undergoing CABG surgery. *Materials and Methods:* This retrospective cohort study was conducted at the Hospital of Lithuania. Inclusion criteria: elective CABG surgery, non-neurocognitive anamnesis, Minimal Mental State Examination score ≥25 before surgery, and age >50. Patients were divided into two groups: DEXA (those who received preoperative dexamethasone 0.1 mg/kg) and non-DEXA (those who did not). Cognitive functions were assessed with the Addenbrooke’s Cognitive Examination test (ACE-III) 7 days post operation. *Results:* The study enrolled 60 patients (DEXA = 30, non-DEXA = 30): male (85%), female (15%). The mean age of the study was 66.1 ± 8.1 and the education was 12 (12–30) years. The groups were similar in the evaluated preoperative characteristics (sex, age, education) (*p* > 0.05). Cognitive impairment (ACE-III score cut–off 88 points) was identified in 40% (n = 12) of participants in the DEXA and 69.3% (n = 21) in the non-DEXA group, with no statistically significant difference between groups (*p* = 0.073). However, the DEXA group had significantly better cognitive scores in attention (Z = 3.145, *p* = 0.002), fluency (Z = 2.25, *p* = 0.024), and spatial ability (Z = 4.444, *p* < 0.001) while language (Z = 1.167, *p* = 0.243) and memory scores (Z = 1.906, *p* = 0.057) showed no significant differences. *Conclusions:* These findings suggest that dexamethasone may provide neuroprotective benefit, reducing postoperative cognitive function domains, such as attention, fluency, and spatial ability, after CABG surgery. Further prospective studies are needed to confirm these findings.

## 1. Introduction

Cardiovascular disease is the leading and largest cause of mortality worldwide [1]. Ischemic heart disease (IHD) alone is the world’s biggest killer, responsible for 8.9 million deaths globally (16% of total deaths), and these numbers are expected to rise over time mainly due to the aging population [2]. Coronary artery bypass grafting (CABG) is a major surgical procedure in which atheromatous blockages in a patient’s coronary arteries are bypassed using harvested venous or arterial conduits [3]. The bypass restores blood flow to the ischemic myocardium, which, in turn, restores function, viability, and relieves anginal symptoms [3]. Approximately 400.000 CABG surgeries are performed annually, making it the most frequently performed major surgical procedure [3].

Although CABG is one of the most commonly performed cardiac surgical procedures to treat coronary artery disease, most patients experience positive changes in their physical health after surgery; however, changes in cognitive function can pose a significant challenge for patients and their loved ones, as they impact daily activities and overall quality of life [4,5]. Cognitive impairment occurs in 40 to 70 percent of patients who undergo CABG surgery with CPB (cardiopulmonary bypass) and persists for a longer period in 30 to 50 percent of these patients. Longer-lasting cognitive impairment adversely affects quality of life and increases the risk of death [6].

The pathophysiology of postoperative cognitive decline (POCD) is complex and still poorly understood. During CPB, despite the use of filters, microemboli (air bubbles, fat, and blood clots) can reach the brain. Additionally, when the aorta is clamped during the procedure, atherosclerotic plaques may dislodge, causing embolism. The study found that patients who died after CABG under CPB conditions had small emboli in their cerebral vessels [7]. Magnetic resonance imaging of the head confirmed that about 50% of CABG patients experience a cerebral microembolic infarction, resulting in mild neurological impairment [8]. It has been confirmed that during CPB, when blood components interact with equipment components, the immune system is activated. This activation triggers widespread systemic inflammatory reactions, leading to reperfusion injury in organs. In addition, peripheral inflammatory factors damage the blood–brain barrier, causing central nervous system (CNS) inflammation and POCD [9]. Inflammation of the brain during cardiac surgery may be the result of a general inflammatory process due to major surgical trauma, CABG, or ischemia and reperfusion injury. Inflammatory mediators can cross the blood–brain barrier directly through active transport mechanisms or indirectly by stimulating the vagus nerve [10]. Cytokines and complement factors acting on the CNS have a detrimental effect on cognitive function [10,11]. Complement activation during surgery can cause further oxidative damage to the CNS, like that seen in dementia. Systemic inflammatory response syndrome, characterized by a pronounced form of leukocytosis, is negatively associated with cognitive function after surgery, especially in the elderly population. Unfortunately, the effects of systemic inflammatory response syndrome can persist for many years after the event [12]. Elevated postoperative IL-6 levels are associated with an increased one-year risk of rehospitalization and mortality among patients who have undergone cardiac surgery [13]. Elevated levels of circulating inflammatory markers and cytokines, including C-reactive protein, IL-1b, IL-6, IL-10, and S-100b, during the perioperative period are associated with POCD [14,15,16]. The long-term effects of CABG surgery on cognitive functions are still not fully understood, emphasizing the need for further research [17,18]. Dexamethasone is a potent synthetic glucocorticoid with strong anti-inflammatory and immunosuppressive properties. It exerts its effects primarily by binding to cytoplasmic glucocorticoid receptors, modulating gene transcription and suppressing the release of proinflammatory cytokines such as IL-6, TNF-α, and CRP. From a pharmacokinetic standpoint, dexamethasone is rapidly absorbed after intravenous administration and has a biological half-life of approximately 36–54 h, allowing for sustained anti-inflammatory action [19]. Dexamethasone can attenuate the systemic inflammatory response induced by cardiopulmonary bypass, reduce endothelial activation, and help preserve myocardial and cerebral function by limiting ischemia–reperfusion injury and metabolic stress [20,21].

Considering the potential neuroprotective properties of dexamethasone and the high incidence of POCD following CABG surgery, this study aimed to assess the effect of perioperative dexamethasone administration on postoperative cognitive outcomes using the ACE-III.

## 2. Materials and Methods

**Figure 1:**
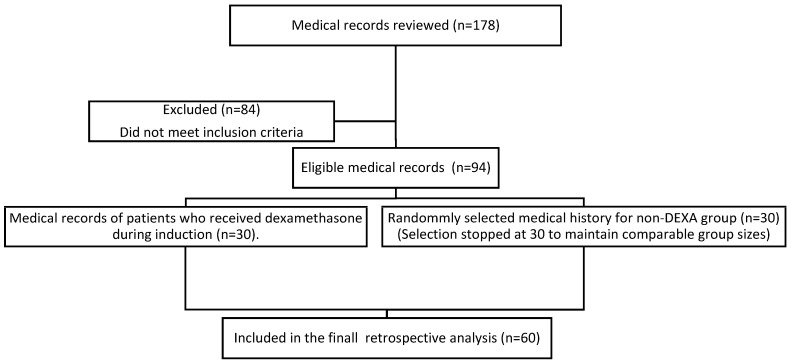
Flow chart of the study.

Permission to conduct the study was obtained from the Bioethics Center of the Lithuanian University of Health Sciences (No. 2023-BEC2-391). The medical records of patients who underwent elective CABG surgery between January 2022 and December 2023 were selected. Anesthesia, CPB, and technic of surgery were standardized according to institutional protocols. Inclusion and exclusion criteria were applied (The criteria are listed below). A total of 60 medical records that met the inclusion and exclusion criteria were included in the study, with 30 (50%) in the DEXA group (0.1 mg per kg dexamethasone was administered during anesthesia induction) and 30 (50%) in the non-DEXA group (dexamethasone was not administered) (Figure 1). A retrospective analysis of medical records was performed.

Inclusion criteria:

1. Elective CABG (under CPB conditions) performed.
2. People aged 50 years or older.
3. No neurological or psychiatric diseases or disorders diagnosed before surgery.
4. MMSE performed before surgery with a score of 25 points or higher. (MMSE served only as a general preoperative cognitive screening tool. Because its domain structure does not align with that of ACE-III, it cannot be used for pre-/post-operative domain-level comparison, although global score correlations with ACE-III were explored.)
5. Cognitive function assessment performed 7–10 days after surgery using the Addenbrooke’s Cognitive Examination (ACE-III).

Exclusion criteria:

1. At least one of the two tests (MMSE or ACE-III) was not performed or was not completed in full.
2. Patients indicated in the data processing consent form that they did not agree to their data being processed for scientific purposes.
3. Regional anesthesia was performed.
4. Patients with uncontrolled diabetes (HbA1c ≥ 7%).
5. Patients had taken medications that affect the CNS (e.g., psychotropic medication)

### Statistical Analysis Methods

Statistical data analysis was performed on a personal computer using the 29.0 Statistical Package for Social Sciences (SPSS) statistical analysis software package. The medical history, Minimal-Mental State Exam (MMSE), and Addenbrooke’s Cognitive Examination (ACE-III) test data were coded and entered in an SPSS 29.0 data file for statistical data processing and analysis.

Using SPSS 29.0, the following calculations were performed: absolute values and percentage expressions of indicators, as well as the level of significance between characteristics. The Shapiro–Wilk test was used to assess the normality of the data. Since the distribution of variables was normal in one group but not in the other, the data were treated as abnormally distributed to ensure a uniform basis for analysis and to perform appropriate intergroup comparisons. When analyzing data for differences between categorical data groups, the Pearson Chi-square (χ^2^) criteria was used. For comparing quantitative data between groups when the data did not meet the normality assumption, the Mann–Whitney U test was used. To assess the dependence of two quantitative variables Spearman’s correlation coefficient (r_s_) was used. A relationship where |r| < 0.3 was considered weak, where 0.3 ≤ |r| < 0.7—moderate, and where |r| ≥ 0.7—strong. When *p* ≤ 0.05, the difference in characteristics between the study groups was considered statistically significant, and when *p* > 0.05, the difference in characteristics between the study groups was considered statistically insignificant.

Descriptive statistics were used. For quantitative indicators that did not meet the assumption of normality, the median, minimum, and maximum values were presented. The characteristics and percentage frequency were used to calculate the frequencies of qualitative indicators. The calculated estimates are presented in tables and diagrams.

## 3. Results

This study analyzed patients’ cognitive functions before (using MMSE as a screening tool) and after surgery (using ACE-III), as well as the correlation between risk factors and ACE-III scores. The median age of patients in the entire sample was 66 years (range 51–84). The patients’ groups (DEXA and non-DEXA) were homogeneous regarding baseline characteristics (Table 1).

The results showed that out of a sample of 60 patients, 29 (48.3%) had POCD based on the ACE-III cutoff <88, while 31 (51.7%) did not. In the DEXA group (n = 30), 18 (60.0%) patients had POCD, whereas the remaining 12 (40.0%) did not. In contrast, in the non-DEXA group (n = 30), 19 (69.3%) patients had POCD, and 11 (36.7%) did not. Although there was no statistically significant difference between the groups, a tendency for POCD to occur less frequently in the dexamethasone group was observed (χ^2^ = 3.270; *p* = 0.071). Positive findings in certain ACE-III subdomains should be interpreted cautiously, as these represent secondary outcomes and multiple comparisons increase the risk of type I error. The differences in the ACE-III total and subcategory scores between the non-DEXA and DEXA groups are evident. Statistically significant differences were found in the ACE-III total score, as well as attention, fluency, and visuospatial scores. All of these scores are higher in the DEXA group compared to the non-DEXA group. The ACE-III scores for both groups are presented in Table 2.

**Figure 2:**
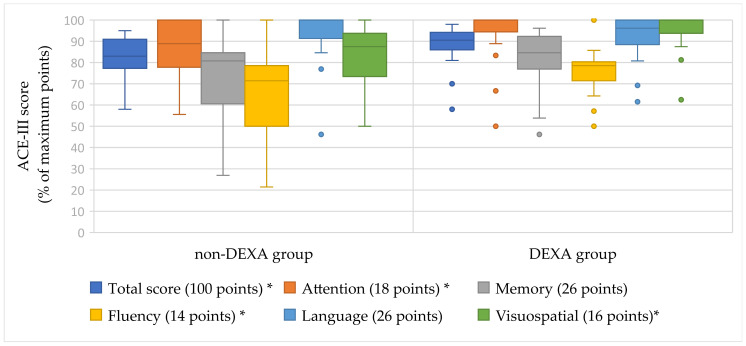
ACE-III total and domain-specific scores (% of maximum points) in the non-DEXA and DEXA groups. Boxes represent the interquartile range (IQR), horizontal lines indicate the median, and whiskers show the minimum and maximum values. Statistical comparisons between groups were performed using the Mann–Whitney U test. * *p* < 0.05.

The Box plot (Figure 2) also clearly shows differences in the scores (expressed as a percentage) between the groups. In all assessed ACE-III scores, the DEXA group demonstrated better cognitive functions compared to the non-DEXA group. The quartile ranges were narrower in the DEXA group, indicating less data variability. Conversely, the quartile ranges in the non-DEXA group were wider. Although there are more outliers in the DEXA group, we can conclude that the medians of the DEXA group’s scores are higher, and the data is more consistent than in the non-DEXA group.

It can be said that the groups are homogeneous regarding risk factors because there were no differences in potential risk factors between the groups, except for CRP (C reactive protein). On the 4th day, CRP was statistically significantly higher in the non-DEXA group than in the DEXA group. The absolute values of the quantitative risk factors in both groups are shown in Table 3.

This study identified risk factors that correlate with ACE-III scores in both DEXA and non-DEXA groups. All significant correlations between ACE-III scores and risk factors, which were statistically different in the groups, are presented in Table 4.

## 4. Discussion

Previous studies have reported conflicting conclusions regarding the effects of glucocorticoids on POCD. For example, Li Qin Li and co-authors conducted a meta-analysis in 2019, which contradicts the results of this study and claims that dexamethasone does not affect POCD or postoperative delirium [22]. However, Li Qin Li’s meta-analysis has limitations, including sample heterogeneity, the selection of only 5 RCTs, the use of inconsistent cognitive function assessment tests, and the examination of three studies on the effect of dexamethasone on cognitive impairment, as well as two studies on postoperative delirium [22].

Five years later, in 2024, researchers from the US, Canada, and India conducted a meta-analysis of RCTs to investigate the effect of corticosteroids on neurocognitive dysfunction. This meta-analysis reviewed 15 RCTs involving more than 15.000 patients. Although the RCTs were selected independently of the type of surgery, 13 RCTs investigated the effect of corticosteroids on cognitive functions after major surgery. The results of this meta-analysis suggest that corticosteroids (methylprednisolone or dexamethasone) reduce the risk of developing POCD. The researchers note that more studies are needed to confirm the effect of prophylactic steroid administration on cognitive function, as the current evidence is insufficient [23]. During this study, a statistically significant difference in CRP was observed between the DEXA and non-DEXA patients, with those prescribed dexamethasone having lower CRP levels. Other studies claim that dexamethasone can modulate the systemic inflammatory response (CRP and other mediators), but clinical results vary—some show a decrease in inflammatory markers, while others do not [24,25].

Upon reviewing the literature, it becomes clear that POCD is influenced not only by CPB or surgery but also by factors like glycocalyx damage. Glycocalyx damage during heart surgery can be caused by hypervolemia, hyperoxia, air microembolism, ischemia and reperfusion, and aging. Damage to the glycocalyx during CBP can cause systemic microcirculation disorders and contribute to postoperative cognitive decline. The cerebral glycocalyx, part of the blood–brain barrier, is particularly sensitive to damage. Protecting it is essential for proper perfusion and neurological health. Glycocalyx damage increases the risk of edema, including cerebral edema [24,25]. As mentioned earlier, CPB significantly impacts the inflammatory response during cardiac surgery when blood components contact the CPB machine [9].

Although we did not investigate the glycocalyx in this study, future research could explore this area. This study found that patients who received higher doses of fentanyl during surgery had higher ACE-III scores. According to Andjela Zivkovic and colleagues, pain during surgery can increase oxidative stress and inflammation, which may contribute to POCD [26]. Stevens describes oxidative stress in the postoperative period as an invisible enemy; hence, anesthesiologists should focus on preventing it [27]. At our clinic, fentanyl is routinely administered in doses according to protocols—repeated doses of 10–20 μg/kg for analgesia. This study also found a weak correlation between serum sodium levels and ACE-III scores. Hyponatremia, characterized by low serum sodium, is linked to cognitive decline in various groups of patients.

Studies show hyponatremia may impair cognitive function, especially in older adults and those with HF or undergoing dialysis. A study of 476 peritoneal dialysis patients indicated that hyponatremia (sodium ≤ 135 mmol/L) was associated with lower cognitive scores, notably on the modified MMSE test. Hyponatremic patients also exhibited poorer cognitive function compared to normonatremic patients [28]. Another study in heart failure patients found that 30% of hyponatremic patients had cognitive impairment, with 92% scoring lower on the Montreal Cognitive Assessment (MoCA) test than normonatremic patients [29]. Hyponatremia was also linked to decreased physical functioning, which can affect overall health. A geriatric study showed that patients with mild to moderate hyponatremia scored lower on all standardized assessments, including those for cognitive function [30].

In summary, hyponatremia is associated with cognitive decline across different patient groups, making serum sodium monitoring crucial after surgery. Anemia is another significant risk factor for POCD. This study observed that lower hemoglobin levels correlated with reduced visuospatial and fluency scores. Guirong Li and colleagues found that anemia is associated with POCD and identified age over 70 and fewer than 6 years of education as independent risk factors. Similarly, our research confirmed that older age and fewer years of education are risk factors for cognitive impairments. Based on the literature, the effects of dexamethasone and other glucocorticoids on POCD and delirium remain uncertain. Although Li Qin Li’s 2019 meta-analysis suggested dexamethasone has no significant effect, larger, more recent meta-analyses indicate that corticosteroids may lower the risk of POCD or delirium [22]. Due to differences in study design, types of surgery, and assessment methods, these findings should be interpreted cautiously. Other factors influencing POCD, such as glycocalyx damage, oxidative stress, electrolyte imbalances, and hyponatremia, also deserve attention. Evidence suggests systemic inflammation can impair cognitive function. While some studies report an increased risk of hyperglycemia with dexamethasone, the differences are often not statistically significant. Further research with larger sample sizes is needed to determine the impact of corticosteroids on postoperative cognitive health reliably.

We also recommend that future studies focus on assessing specific domains of cognitive function rather than relying solely on the overall test score. In our study, no significant difference was found in the total ACE-III score between the groups; however, when individual cognitive domains were analyzed separately, significant differences appeared. This emphasizes the importance of evaluating aspects of cognition—such as attention, fluency, and visuospatial skills—because global scores can mask subtle yet clinically important changes in specific areas. Additionally, our findings suggest that dexamethasone may help reduce POCD in certain cognitive domains, which could lead to a lower overall incidence of POCD after cardiac surgery.

## 5. Conclusions

Patients receiving 0.1 mg/kg dexamethasone during anesthesia were less likely to experience cognitive impairment (40.0% vs. 69.3% in the non-DEXA group). They scored better on ACE-III tests, particularly in the attention, fluency, and visuospatial subcategories. No difference was seen in language and memory scores. In the dexamethasone group, lower postoperative sodium, older age, and lower fentanyl doses linked to poorer cognition, while age and Hb level were key risk factors in the non-DEXA group.

The data from this study suggest that administering dexamethasone during anesthesia induction may be linked to improved postoperative cognitive outcomes. Therefore, this intervention may be associated with more favorable postoperative performance in certain cognitive domains, although these findings should be interpreted cautiously due to the lack of comparable preoperative domain-level assessment and require confirmation in prospective studies.

### Limitations of the Study

Retrospective Single-center design study—medical records do not contain data on the patients’ lifestyle—alcohol consumption, diet, physical activity, leisure activities, nature of work, etc.—and may introduce selection bias and restrict the generalizability of the findings, as patient characteristics and clinical practices of one institution may not fully represent broader populations or settings.

Small study sample (n = 60)—limited number of medical records meeting the selection criteria. Given this modest sample size and borderline *p*-values for the primary outcome, there is a risk of type II error, meaning the study may be underpowered to detect true differences. Additionally, multiple analyses of secondary ACE-III subdomains increase the risk of type I error, so these findings should be interpreted cautiously.

Short follow-up (7–10 days)—restricts the ability to assess longer-term outcomes, potential delayed complications, or sustained treatment effects.

MMSE was assessed before surgery, rather than ACE-III, which was used after surgery—these instruments differ in structure and subdomain composition, making them unsuitable for direct domain-level comparison. Therefore, although correlations between MMSE and ACE-III global scores were examined, they do not allow interpretation of true pre-/post-operative domain-specific change. The study is thus limited to postoperative cross-sectional comparisons between the DEXA and non-DEXA groups.

## Figures and Tables

**Table 1:** Preoperative characteristics in groups.

Characteristics	DEXA Group (n = 30)	Non-DEXA Group (n = 30)	*p*-Value
**Male (n, %)**	26 (86.7)	25 (8.3)	0.718 *
**Female (n, %)**	4 (13.3)	5 (16.7)	
**Age (years)**	65 (52–82)	68 (51–84)	0.801 **
**Duration of education (years) median (range)**	12 (7–30)	12 (9–20)	0.214 **
**MMSE score median (range)**	28 (25–30)	28 (25–30)	0.660 **

* Chi-square test was performed. ** Mann–Whitney U test was performed.

**Table 2:** ACE-III score distribution in subcategories, DEXA group (n = 30) and non-DEXA group (n = 30).

ACE-III Subcategories	DEXA Group (n = 30) Median (Range)	Non-DEXA Group (n = 30) Median (Range)	*p*-Value *
**ACE-III total score (max = 100)**	90.5 (58–98)	83 (58–95)	0.002
**Attention (max = 18)**	18 (9–18)	16 (10–18)	0.002
**Memory (max = 26)**	22 (15–25)	21 (7–26)	0.057
**Fluency (max = 14)**	11 (7–14)	10 (3–14)	0.024
**Language (max = 26)**	26 (16–26)	25 (12–26)	0.243
**Visuospatial (max = 16)**	16 (10–16)	14 (8–16)	<0.001

* Mann–Whitney U test.

**Table 3:** Quantitative factors for the DEXA (n = 30) and non-DEXA groups (n = 30).

Risk Factors	DEXA Group	Non-DEXA Group	*p*-Value ***
	Median (Range)	Median (Range)	
**Age**	65 (52–82)	68 (51–84)	0.801
**Duration of education (years)**	12 (7–30)	12 (9–20)	0.214
**MMSE score before surgery**	28 (25–30)	28 (25–30)	0.515
**Duration of hospitalization (days)**	15.5 (10–33)	16 (10–39)	0.836
**Dose of Fentanyl during surgery μg/kg**	10.1 (3.97–17.54)	8.23 (5.26–13.33)	0.076
Blood pH *	7.422 (7.324–7.522)	7.424 (7.273–7.486)	0.679
Potassium *	4.1 (3.5–5.3)	4.0 (3.6–5.8)	0.766
Sodium *	140 (136–145)	140.5 (133–146)	0.794
Glycemia *	6.1 (5.3–8.9)	6.0 (4.3–9.9)	0.773
Lactates *	0.85 (0.5–1.9)	0.9 (0.5–3.8)	0.864
Hemoglobin *	143.5 (101–183)	139.5 (78–171)	0.383
Blood pH **	7.429 (7.352–7.476)	7.438 (7.340–7.488)	0.673
Potassium **	4.3 (3.8–4.7)	4.3 (3.7–5.1)	0.564
Sodium **	138 (133–142)	138 (132–143)	0.840
Glycemia **	6.4 (5.2–10.2)	6.95 (5.3–14.4)	0.206
Lactates **	0.6 (0.4–10.0)	0.6 (0.4–11.0)	0.356
Hemoglobin **	101 (78–142)	96.5 (73–112)	0.107
**C reactive protein (in 4th day after surgery)**	122 (21–273)	168 (72–330)	0.005
**Body mass index**	27.7 (18.72–38.89)	27.73 (18.11–37.58)	0.367
**Duration of surgery (minutes)**	182.5 (145–360)	180 (125–330)	0.547
**Duration of cardiopulmonary bypass (minutes)**	83.5 (61–125)	83.5 (56–166)	0.813
**Duration of aorta clamping (minutes)**	41 (25–75)	39.5 (24–76)	0.534

* Checked before anesthesia induction. ** Checked 3 days after anesthesia induction. *** Mann–Whitney U test.

**Table 4:** Correlation between ACE-III scores and quantitative risk factors in DEXA and non-DEXA group (n = 30).

ACE-III Subcategories	Risk Factors	DEXA Group		Non-DEXA Group	
		Correlation Coefficient (r_s_)	*p*-Value	Correlation Coefficient (r_s_)	*p*-Value
**Total ACE-III score**	Age	−0.554	<0.001	−0.327	0.039
	Sodium **	0.410	0.012	-	0.870
	Dose of fentanyl during surgery	0.396	0.015	-	0.247
	Duration of education	0.448	0.007	-	0.489
	MMSE score	0.479	0.004	-	0.694
**Attention**	MMSE score	0.407	0.013	-	0.272
	Sodium **	0.454	0.006	-	0.5
	Duration of surgery	-	0.486	−0.342	0.032
	Potassium **	-	0.677	−0.307	0.043
**Fluency**	Age	0.668	<0.001	-	0.117
	MMSE score	0.547	<0.001	-	0.392
	Hemoglobin *	0.358	0.026	-	0.214
	Sodium **	0.371	0.022	-	0.231
	Duration of CPB	0.352	0.028	-	0.414
	Potassium *	-	0.111	−0.348	0.03
	Glycemia **	-	0.479	−0.383	0.018
	Duration of education	0.355	0.27	-	0.219
	Dose of fentanyl during surgery	0.412	0.012	-	0.235
**Visuospatial**	Age	−0.661	<0.001	-	0.103
	MMSE score	0.307	0.049	-	0.284
	Dose of fentanyl during surgery	0.412	0.012	-	0.432
	Hemoglobin *	0.447	0.007	0.394	0.016
	Glycemia *	−0.318	0.044	-	0.268
	Potassium **	-	0.355	−0.354	0.027
	Duration of education	0.320	0.042	-	0.138
	Hemoglobin **	-	0.211	0.342	0.032

* Checked before anesthesia induction. ** Checked 3 days after anesthesia induction. -: No correlation was found. r_s_: Spearman correlation.

## Data Availability

The data presented in this study are available upon request from the corresponding author.

## Abbreviations

The following abbreviations are used in this manuscript:

ACE-III	Addenbrooke’s cognitive examination—III
ASA	American society of Anesthesiologists Physical Status Classification System
CABG	Coronary artery bypass grafting
CPB	Cardiopulmonary bypass
CRP	C reactive protein
Hb	hemoglobin
IHD	Ischemic heart disease
LUHS	Lithuanian University of Health Science
MMSE	Minimal-Mental State Exam
p	level of statistical significance
POCD	Post Operative Cognitive Decline
r_s_	Spearman correlation
